# pH induced reversible assembly of DNA wrapped carbon nanotubes

**DOI:** 10.1186/1752-153X-7-14

**Published:** 2013-01-24

**Authors:** Ying Wan, Gang Liu, Xinhua Zhu, Yan Su

**Affiliations:** 1 School of Mechanical Engineering, Nanjing University of Science and Technology, Nanjing, Jiangsu 210094, Peoples Republic China; 2 Shanghai Institute of Measurement and Testing Technology, Shanghai 201203, Peoples Republic China

**Keywords:** pH controlled reversible assembly, Single walled carbon nanotubes (SWCNTs), i-motif, G-quadruplex

## Abstract

**Background:**

Reversible assembly and disassembly of nanostructures has important function in controllable construction of nanodevices. There are several methods to achieve reversible assembly/disassembly, such as pH, temperature, DNA hybridization and so on. Among these methods, pH driven reversible assembly presents superiority due to its ease-of-use and no waste produced. Herein we report a novel design that use two single-stranded (ss) DNAs wrapped single walled carbon nanotubes (SWCNTs) for the pH controlled assembly of SWCNTs without generation of waste.

**Results:**

Both of the two DNAs with a same wrapping sequence of d(GT)20 and different free terminals showed a very high tendency to wrap around carbon nanotubes. The assembly was driven by the hybridization between the two free terminals of wrapped DNAs on the neighboring SWCNTs: i-motif (four-stranded C-quadruplex) and its complemental stranded G-quadruplex which would form tight tetraplexes and break the hybridization under slightly acidic conditions. Thus the assembly and disassembly are reversibly controlled by pH. And this assembly/disassembly process can be easily distinguished by naked eyes. Gel electrophoresis and Atomic Force Microscope are used to demonstrate the assembly and disassembly of SWCNTs at different pH.

**Conclusions:**

A novel pH induced reversible assembly and disassembly of SWCNTs was realized which may have potential applications in the area of controlled assembly of nanostructures.

## Background

Nanostructure has potential applications in future fabricating and nanodevices [1-8]. “Bottom up” construction of exquisite nanostructure based on different kinds of nanomaterials has attracted numerous attentions [9-13]. Several studies are particularly devoted to nanostructure based on DNA and other nanomaterials such as gold nanoparticles [14,15], carbon nanotubes [16,17], graphene [18] and so on [19,20]. Nanostructures formed by single-walled carbon nanotubes (SWCNTs) and DNA combine both the size-dependent properties of the SWCNT and the molecular recognition and biological function of DNA, which have potential applications in molecular electronics [21,22] and biomedical engineering [23-25]. Both covalent [26] and noncovalent [27] associations are used to construct SWCNT–DNA complexes. However, self-assembly strategies based on the biorecognition capability of single-stranded DNA (ssDNA) have been proposed to be a promising one [16,25].

Reversible assembly and disassembly of nanostructures has important function in controllable construction of nanodevices [28-30]. There are several methods to achieve reversible assembly/disassembly, such as pH, temperature, DNA hybridization and so on. Deng and co-workers demonstrated reversible assembly/disassembly of DNA–SWCNT conjugates switched by DNA hybridization [31]. However, changes in pH offered a simple but versatile way to control the assembly of materials. Qu et al. reported a duplex-based DNA-SWCNT self-assembled nanostructure based on a four-stranded DNA structure, G-quadruplex, and i-motif DNA [32]. This driven mechanism is reversibly controlled by pH, but still need complex covalent modification of SWCNT.

Herein we report a pH driven reversible assembly of DNA wrapped SWCNTs. SWCNTs were wrapped by two single strand DNAs which differed at their terminal: one had an i-motif sequence and the other had its complemental sequence G-quadruplex. Both of the DNAs with a wrapping sequence of d(GT)20 had a very high tendency to wrap around carbon nanotubes and made the SWCNTs stably water dispersed. However, The SWCNTs formed conjugates as the two DNA terminals hybridized to each other under slightly basic conditions, and they were disassembled under slightly acid conditions as the tight G-quadruplex and i-motif DNA structure formed. Thus the design of a reversible SWCNTs nanostructure driven by pH changes realized.

## Results and discussion

### The strategy

**Figure 1:**
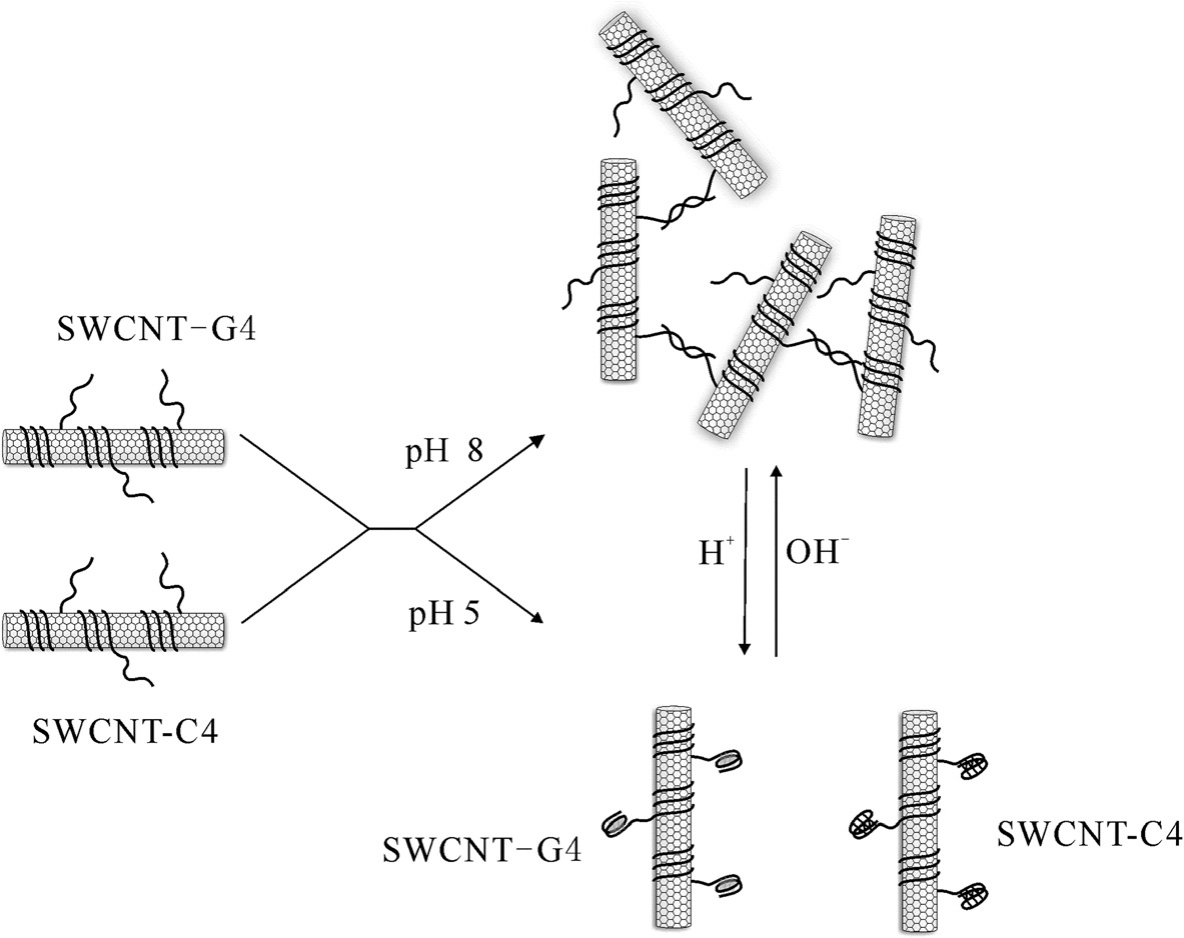
**Strategy of pH induced reversible assemble of DNA wrapped SWCNTs.** The mixture of Two SWCNT-DNA conjugates (SWCNT-G4 and SWCNT-C4) formed 3D aggregates at pH 8.0 through hybridization of G4 and C4. While the pH of buffer solution was adjusted to 5.0, G4 and C4 are formed and SWCNTs were dispersed. This assembly and disassembly process was reversible by adjusting the pH. Sequence of C4 DNA:(GT)20 –TTT TTT TTT TCC CAA TCC CAA TCC CAA TCC C. Sequence of G4 DNA: (GT)20 –TTT TTT TTT TGT GAT TGT GAT TGT GAT TGT G.

SWCNTs were wrapped by two single-strand DNAs which had a same wrapping sequence of d(GT)_20 _and different free terminals respectively (Figure 1). One of the free terminals contains G-quadruplex (G4), and the other contains i-motif (C4) in their sequences. A single-base mutation was added to the G-quadruplex domain to prevent the nonspecific SWCNTs assembly caused by the formation of the interparticle G-quadruplex. As mentioned in literatures, this sequences were proved to be useful in making other DNA based nanodevices [33,34]. Under basic conditions, C4 hybridized with its complementary G4, therefore SWCNTs formed a net-shape conjugates. When the pH was decreased to 5.0, C4 and G4 formed intramolecular i-motif and G-quadruplex tetraplex respectively, so the SWCNTs do not aggregate. If the pH was adjusted to 8.0 again, the net-shape conjugate reformed. The assembly and disassembly process was reversible as the pH reversed. As a result, the pH actuated reversible SWCNTs assembly was formed.

### SWCNTs at slightly basic solution and slightly acid solution

**Figure 2:**
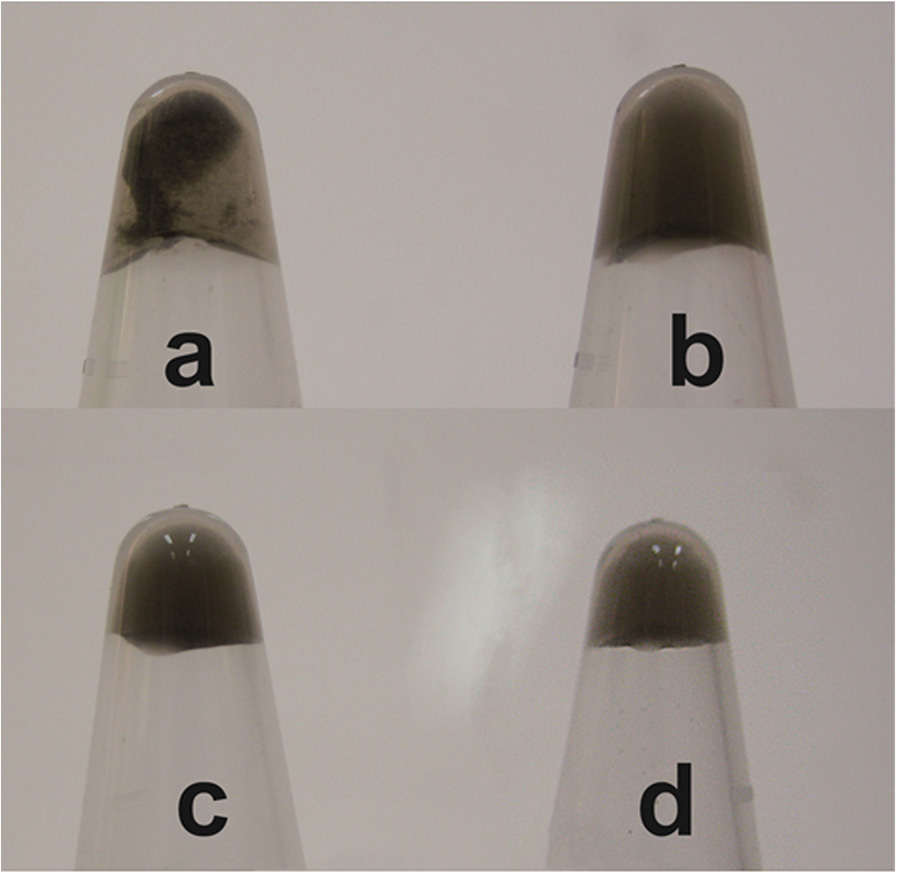
**Photograph of the two vials containing SWCNT-G4 and SWCNT-C4 in pH 8.0 (a) and pH 5.0 (b), and two vials containing only SWCNT-G4 in pH 8.0 (c) and pH 5.0 (d).** After staying at room temperature for 30 min, Centrifugations at 2000 g for 10 s were applied to the two vials.

The mixtures of SWCNT-G4 and SWCNT-C4 in stoichiometric equivalents were adjusted to pH 8.0 (Figure 2a) and pH 5.0 (Figure 2b) respectively. After a 25-min incubation and a short centrifugation at 2000 g for 10 s, these two vials were observed by naked eye. Precipitation was seen at pH 8.0 which was respected to the conjugation of SWCNTs, while the solution was homogeneous at pH 5.0 showing that SWCNTs were dispersed well. As a contrast, two other vials of SWCNT-G4 only were adjusted to pH 8.0 (Figure 2c) and pH 5.0 (Figure 2d) respectively and observed after the same treatment. Interestingly, both of them were dispersed well. This phenomenon proved the specific SWCNTs conjugation was not affected by the change of pH condition.

### Reversible assembly of SWCNT by adjusting pH

**Figure 3:**
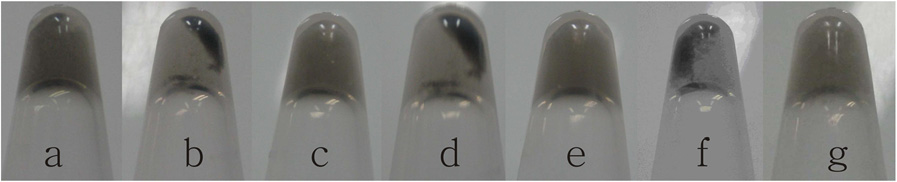
**Reversible assembly and disassembly of SWCNTs by adjusting pH between 5.0 and 8.0.** Solutions in tubes **a**, **c**, **e**, and **g** are at pH 5.0, and tubes **b**, **d**, and **f** are at pH 8.0. Three cycles of assembly and disassembly of SWCNTs by pH switching are demonstrated here. Centrifugations at 2000 g for 10 s were applied after each pH changing to make the aggregates more visually discernible.

Two SWCNTs (SWCNT-G4 and SWCNT-C4) were mixed in stoichiometric equivalents in a vial. Then the pH was adjusted to 5.0 and 8.0 sequently and this process was cycled several times. As was seen in Figure 3, precipitate was seen when pH was changed to 8.0 while no precipitate was seen at pH 5.0. And the deposition and re-dispersion of the precipitate was reversible when the pH reversed.

### Gel electrophoresis and atomic force microscopy (AFM) illustration

Agarose gel electrophoresis and AFM were employed to interrogate the assembly and disassembly extent of SWCNT–C4/SWCNT–G4 at both acidic and basic conditions.

**Figure 4:**
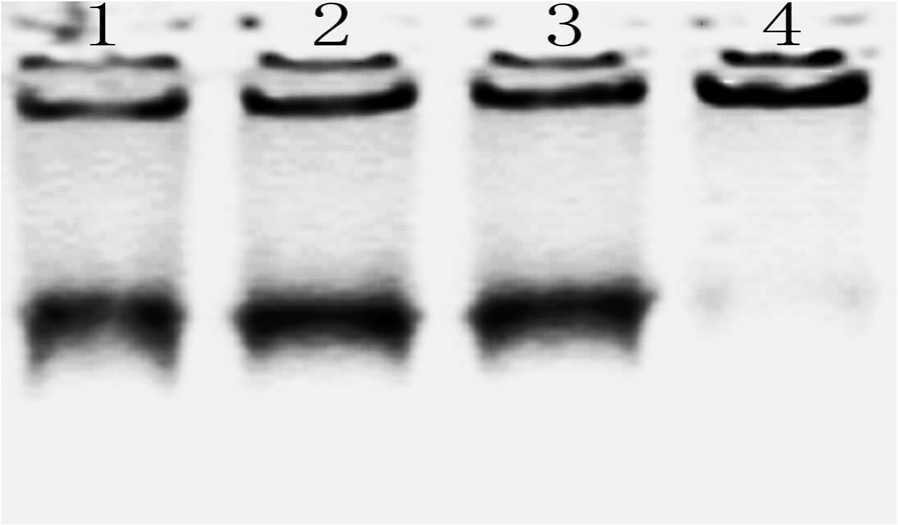
0.5% agarose gel electrophoresis of the SWCNT-G4 (lanes 1), SWCNT-C4 (lanes 2), asswbly of SWCNT-G4 and SWCNT-C4 aggregates in pH 5.0 (lanes 3) and pH 8.0(lanes 4).

Gel electrophoresis was employed to characterize the migration status of SWCNTs at different conditions (Figure 4). SWCNT-G4 (lane 1) and SWCNT-G4 (lane 2) could migrate out of the well as a single band. The mixture of two SWCNTs in pH 5.0 (lane 3) and 8.0 (lane 4) showed different migration status: When the pH was adjusted to 5.0, the mixture could also migrate out of the well and the speed was the same as SWCNT-G4 and SWCNT-C4, which suggeted that the i-motif and G-quadruplex were formed and SWCNTs dispered well. While the pH of the mixture was adjusted to 8.0, the aggregation of the SWCNTs could not penetrate into a 0.5% agarose gel but stayed in the well, which suggested that that SWCNT-C4 and SWCNT-G4 hybridized with each other and crosslinked SWCNTs was formed.

**Figure 5:**
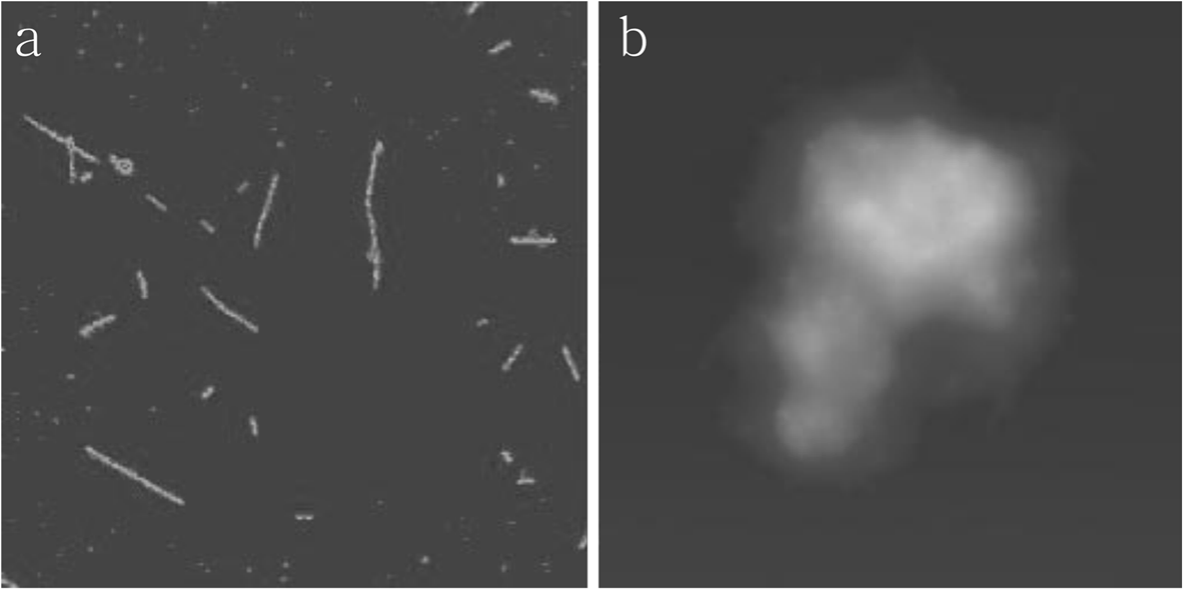
AFM images of the conjugation of SWCNT-G4 and SWCNT-C4 at pH 5.0 (left) and pH 8.0 (right).

The SWCNT-DNA aggregation controlled by pH was further checked by AFM. A centrifugation-assisted precipitation was employed to visually observe the dispersion and the precipitate of SWCNTs mixture. As seen in Figure 5, the mixture of SWCNTs despersed well and uniformly at pH 5.0 and formed a big area of aggregate at pH 8.0. This result further confirmed the result in gel electrophoresis which reveals the sharp contrast between the dispersed and aggregated states of the DNA–SWCNT conjugates.

### The kinatic of aggregation

**Figure 6:**
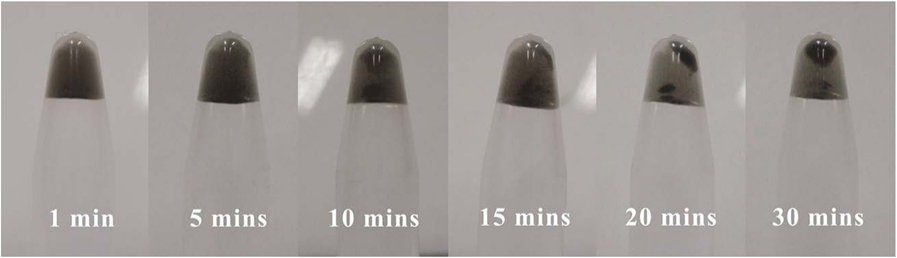
Photograph of assembly of SWCNTs at different time.

To investigate the kinetic of SWCNTs assembly, the pH of mixture of SWCNT-G4 and SWCNT-C4 was adjusted to 8.0. The solution was incubated at room temperature and was observed by a naked eye. A centrifugation-assisted precipitation was adoped at certain points and photographs were taken. The precipitation process was shown in Figure 6. There was little deposit after 5 min when the pH was adjusted to 8.0. Obvious conjugates were seen after 10 min and supernatant was clear after 30 min. it was suggested that the assembly of SWCNTs reached saturation point after 30 min.

## Experimental

### Synthesis of DNA wrapped SWCNT

DNA oligonucleotides were purchased from Sangon Inc. (Shanghai, China) with their sequences listed in Figure 1. A mutation of the G-quadruplex domain with a single base (from GGG to GTG) will disrupt the formation of the interparticle G-quadruplex and prevent the SWCNTs assembly. DNA wrapped SWCNT was prepared following a reported process. In briefly, 100 μL of aqueous solution containing about 0.1 mg of SWCNTs (Sigma Chemicals), 0.025 mg G4 or C4, and 0.1 M NaCl was sonicated at a power of about 4 W using a VC130PB probe-type sonicator (Sonics materials inc.). The whole sonication process was incubated in an ice-water bath. Then free DNA was removed using centrifugation. Before centrifugation, MgCl_2_ was added to the DNA and SWCNT mixture with the concentration of 30 mM to help precipitation of the SWCNT/DNA conjugates from the solution. A centrifugation at 2000 g was taken for 2 min. The supernatant solution was carefully removed using a pipette tip. The resulted DNA/SWCNT conjugate was redispersed in 0.5xTBE buffer (Tris, 44.5 mM; EDTA, 1 mM; and boric acid, 44.5 mM, pH 8.0) containing 30 mM NaCl plus 10 mM extra EDTA to complex with residual Mg^2+^ in the precipitate. This step was cycled several times.

### pH driven reversible SWCNT-DNA assembly

SWCNT-G4 and SWCNT-C4 were mixed in stoichiometric equivalents in a vial. Then the pH was adjusted to 5.0 by adding 1 M HCl, and then the solution was incubated for 30 min at 25°C. The resulted mixture was centrifuged at 2000 g for 30 s and observed. Then the pH was adjusted to 8.0 by adding NaOH and the solution was treated same as pH 5.0. The process was repeated several times. Assembled and disassembly products were both checked by gel electrophoresis or AFM.

### Kinetic of the assembly of two SWCNTs

SWCNT-G4 and SWCNT-C4 were mixed in stoichiometric equivalents in a vial and the pH was adjusted to 8.0. Then the solution was incubated at 25°C and observed at different time.

### Agarose gel electrophoresis

SWCNT-DNA conjugates were loaded into 0.5% agarose gel and run in 0.5 × TBE at 10 V/cm. The DNA-hybridization leaded to the SWCNT aggregates which could not run into the 0.5% agarose gel and was held as black deposit in the gel-loading wells.

### AFM analyses

AFM images were recorded using a Nanoscope IIIa apparatus (Digital Instruments, USA) equipped with a J Scanner. A droplet of SWCNTs mixture sample was cast onto a freshly cleaved mica surface, followed by drying at room temperature.

## Conclusion

In summary, we provided a pH controlled reversible assembly of SWCNTs. SWCNTs were wrapped by two ssDNAs which contained wrapped sequence and different free terminals. This assembly was driven by the hybridization between their free terminals: i-motif and G-quadruplex. A mutation of the G-quadruplex domain with a single base (from GGG to GTG) was used to disrupt the formation of the interparticle G-quadruplex and prevent the nonspecific SWCNTs assembly. As i-motif and G-quadruplex were both formed at slightly acidic conditions, the assembly was controlled by pH and reversible. This assembly/disassembly process was easy to control without generation of waste and accompanied by precipitation that was clearly visible to the naked eye. This system may develop into a fast, highly reversible pH-sensitive device that may have potential applications in the area of nanobiotechnology. For example, this pH induced controllable assembly of SWCNTs can offer a potential method to desired novel pH-sensitive multifunctional architectures and/or biosensing devices.

## Abbreviations

ssDNA: Single-stranded DNA; SWCNT: Single walled carbon nanotube; C4: Four-stranded C-quadruplex; G4: Four-stranded G-quadruplex.

## Competing interests

The authors declare that they have no competing interests.

## Authors’ contribution

YW proposed the subject, designed the study, participated in the results discussion and carried out the synthesis of DNA wrapped SWCNT. GL carried out the agarose gel electrophoresis and Atomic Force Microscopy Analyses and helped to draft the manuscript. XZ and YS conceived of the study, and participated in its design and coordination and helped to draft the manuscript. All authors read and approved the final manuscript.
